# Longitudinal analysis of hippocampal subfield atrophy and network centrality associated with cognitive decline in Alzheimer's disease progression

**DOI:** 10.1002/mp.70379

**Published:** 2026-03-03

**Authors:** Sewon Lim, Youngjin Lee

**Affiliations:** ^1^ Department of Health Science General Graduate School of Gachon University Yeonsu‐gu Incheon Republic of Korea; ^2^ Department of Radiological Science Gachon University Yeonsu‐gu Incheon Republic of Korea

**Keywords:** Alzheimer's disease progression, hippocampal subfields, network‐based analysis

## Abstract

**Background:**

Alzheimer's disease (AD) is a progressive neurodegenerative disorder characterized by structural degeneration of the hippocampus. Previous studies have demonstrated that specific hippocampal subfields, such as the cornu ammonis (CA)1 and the subiculum, are susceptible to AD‐related atrophy. However, most previous studies have focused on cross‐sectional volumetric changes without investigating the interrelationships between subfields or their network‐level functions throughout the disease progression.

**Purpose:**

To examine the longitudinal volumetric changes in the hippocampal subfields over 2 years in individuals who progressed from mild cognitive impairment (MCI) to AD. In addition, we aimed to investigate the associations between cognitive decline, inter‐region structural correlation, and network‐based centrality profiles of subfields based on atrophy covariance and changes in subfield volume.

**Methods:**

T1‐weighted magnetic resonance images of 258 participants who progressed from MCI to AD were obtained from the Alzheimer's Disease Neuroimaging Initiative. Hippocampal subfield volumes were extracted at baseline and during follow‐up using FreeSurfer's longitudinal pipeline (v7.4.1). The subfield volume changes were examined using the paired‐*t* tests. Cognitive decline was measured as the percentage change in the Mini‐Mental State Examination (MMSE) scores. The partial Pearson's correlations between subfield volume changes and MMSE change were calculated after adjusting for baseline age, sex, education level, and apolipoprotein ε4 status. A structural covariance network was constructed using inter‐subfield partial correlations. Four graph‐theoretical centrality measurements (degree, betweenness, closeness, and eigenvector) were computed from the network to identify structurally central subfields.

**Results:**

Most hippocampal subfields demonstrated important volume decreases over 2 years, with the left fimbria, subiculum head, and dentate gyrus head showing the most atrophy. The left hippocampus showed significantly greater volume decreases than the right hippocampus. Volume changes in the left presubiculum body, the CA3 head, and the dentate gyrus head were strongly correlated with MMSE decline. Notably, structured covariance patterns between anatomically and functionally relevant subfields within the CA1‐CA3‐CA4‐dentate gyrus axis and subiculum complex were found by inter‐regional analysis. Network‐based analysis identified the left CA1 head and left dentate gyrus head as central hubs across all four‐centrality metrics. Other subfields, including the left subiculum head and left molecular layer head, also showed high centrality in several respects, indicating their possible coordinating functions in hippocampal degeneration.

**Conclusions:**

This study provides a comprehensive longitudinal analysis of hippocampal subfield atrophy, inter‐regional co‐atrophy patterns, and network centrality during MCI‐to‐AD progression. Our findings demonstrate that several subfields, including the left CA1 and dentate gyrus, are structurally and functionally central in the hippocampal atrophy network. The integration of volumetric, correlation‐based, and graph theory‐based approaches offers new insights into the coordinated degeneration of the hippocampus in AD, emphasizing the importance of subfield‐level network dynamics in understanding the disease progression.

## 1 INTRODUCTION

Alzheimer's disease (AD) is a progressive neurodegenerative disorder characterized by memory loss and cognitive impairment, ultimately leading to the loss of independence in daily life.^1^ In the typical progression of AD, neuronal loss starts in the medial temporal lobe, particularly the hippocampus, and spreads to higher‐order and associative cortices.^2^ The hippocampus is one of the primary brain regions affected in AD, and the dysfunction of the hippocampus leads to memory impairment.^3^ The hippocampus is a crucial brain structure involved in episodic memory and spatial navigation.^4^ Hippocampal atrophy is one of the earliest macroscopic characteristics of AD consistently observed in neuropathological and neuroimaging studies.^5^ The hippocampus is not a homogeneous structure but it can be divided into various subregions anatomically and functionally, with different connectivity to other brain regions and varying susceptibility to diseases.^6^ The hippocampus comprises several subfields, including the Cornu Ammonis (CA) 1–4, the dentate gyrus, and the subiculum.^7^ Each subfield of the hippocampus plays a distinctive role in memory processing. For example, CA1 and the subiculum are related to episodic memory consolidation and retrieval, while the dentate gyrus and CA3 are involved in encoding new information.^8^ A major pathological feature of AD is the formation of neurofibrillary tangles (NFT), which initially affect the entorhinal cortex and subsequently spread through the CA1, subiculum, and other hippocampal subfield.^9^ Therefore, analyzing the entire hippocampus as a single region can obscure these regionally specific atrophy patterns.

With advances in high‐resolution magnetic resonance image (MRI) and segmentation techniques, recent studies have increasingly focused on subfield‐level hippocampal atrophy rather than whole‐structure analysis.^6^, ^10^, ^11^, ^12^, ^13^ Previous studies have demonstrated that subfields, such as CA1 and the subiculum, are primarily degenerated in mild cognitive impairment (MCI) and AD compared to cognitively normal controls.^14^, ^15^, ^16^ Moreover, the atrophy of hippocampal subregions is closely associated with the progression of AD and cognitive decline.^6^, ^7^, ^17^, ^18^, ^19^ In particular, longitudinal studies have revealed that volume reductions in subfields such as CA1, the molecular layer, and the subiculum correlate with cognitive deterioration.^19^ These findings suggest that analyzing the hippocampus at the subfield level can provide sensitive biomarkers for AD.

Nonetheless, most existing studies have primarily focused on the relationship between individual subfield atrophy and cognitive function, with limited attention to inter‐subfield interactions or network‐level analyses. In recent neuroscience research, network‐based approaches that account for structural and functional interconnectivity are gaining prominence.^20^, ^21^, ^22^, ^23^, ^24^

Structural volume covariance network analyses have been widely used to characterize coordinated patterns of regional brain atrophy and network‐level vulnerability in AD.^25^, ^26^, ^27^ In particular, the subfields of the hippocampus are closely interconnected through anatomical and functional pathways, functioning as a complex system that performs integrated memory processing, which enhances the validity of the network approach.^28^, ^29^, ^30^ Accordingly, interpreting the structural changes between subfields from a network perspective can provide novel insights into inter‐regional interactions and system‐level pathological patterns that are not apparent in conventional region‐wise analysis.

Therefore, in this study, we aimed to quantitatively analyze the longitudinal volume changes in the hippocampal subfields of patients who progressed from MCI to AD over 2 years. We compared the subfield volumes at baseline and follow‐up to identify region‐specific atrophic changes associated with disease progression. Furthermore, we examined the relationship between these volumetric changes and variations in cognitive scores. In addition, by analyzing the correlation patterns of volume changes between subfields, we explored the interregional structural relationships within the hippocampus, constructed a network based on this, and calculated centrality indices to identify the subfields that play a central role in the progression of AD.

## 2 METHODS

### 2.1 Study participants

**FIGURE 1:**
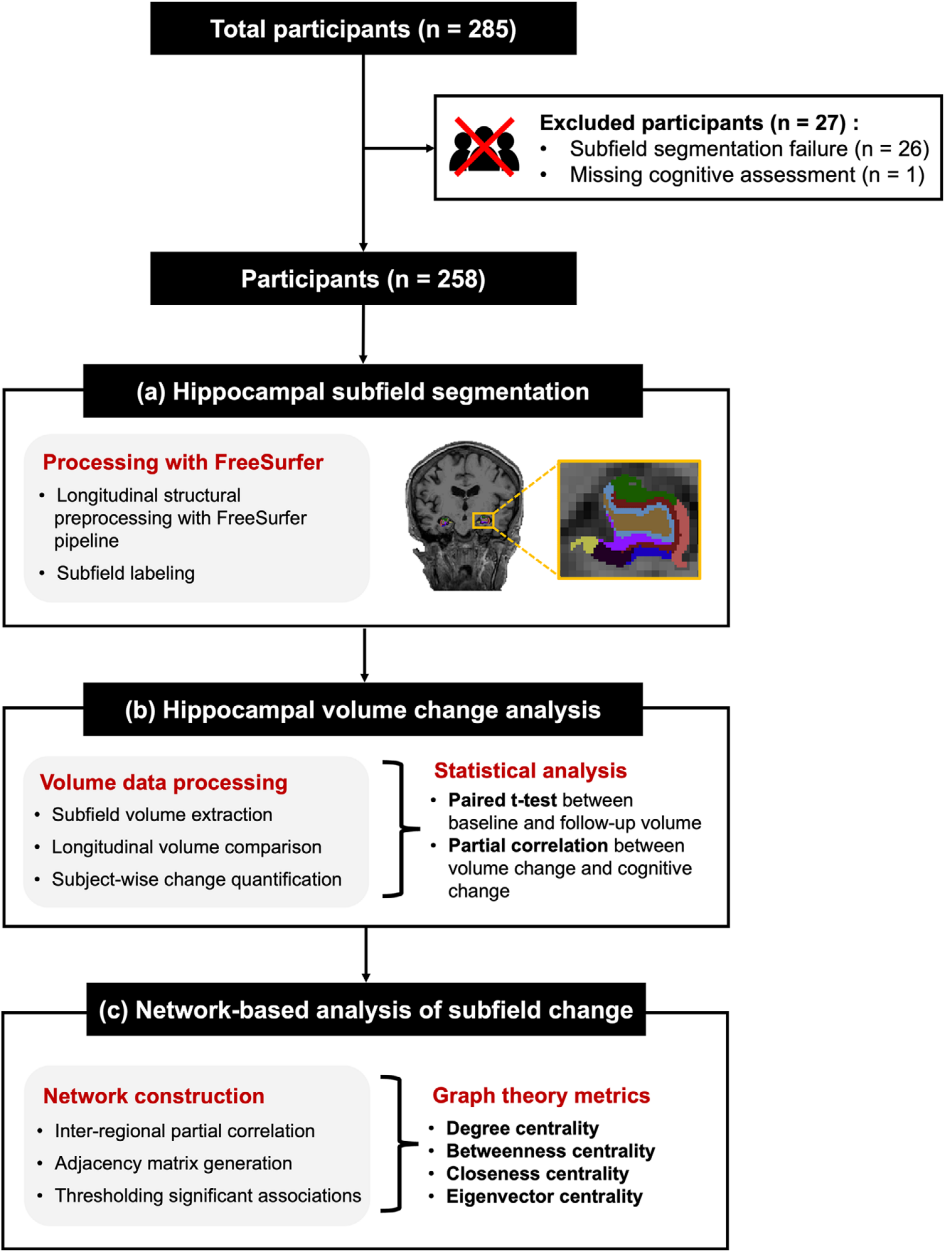
Flow chart of the overall study: (a) Hippocampal subfield segmentation, (b) Hippocampal volume change analysis, and (c) Network‐based analysis of subfield change.

All data used in this study were obtained from the Alzheimer's Disease Neuroimaging Initiative (ADNI) database (adni.loni.usc.edu) in November 2025, by querying the entire ADNI project without restriction to a specific study phase. We included patients who had undergone T1‐weighted structural MRI scans at baseline and the 2‐year follow‐up, along with their cognitive assessments and demographic information. Figure 1 provides an overview of the study design. Of the 285 participants who progressed from MCI to AD within 2 years, we excluded if hippocampal subfield segmentation failed to yield valid quantitative outputs, or missing cognitive assessment data. Finally, this study included 258 participants (age 75.10 ± 7.17 years; 102 [39.53%] females). Participants with two apolipoprotein E (APOE) ε4 alleles were categorized as (+/+), those with one ε4 allele as (+/−), and those without any ε4 alleles as (−/−). This study was conducted in accordance with the Declaration of Helsinki and was approved by the Institutional Review Board of Gachon University (1044396‐202405‐HR‐086‐01).

### 2.2 Neuropsychological assessment

Cognitive function was assessed using the Mini‐Mental State Examination (MMSE). The MMSE is a standardized screening tool that evaluates multiple cognitive domains, including memory, attention, language, visuospatial skills, and executive functioning.^31^ Participants were classified as having MCI at baseline and progressed to AD at the 2‐year follow‐up, based on their MMSE scores in accordance with the ADNI criteria (https://adni.loni.usc.edu/methods/documents/). Cognitive decline was determined by the changes in the MMSE scores over the 2‐year period: cognitive decline (%) = (MMSE at follow‐up—MMSE at baseline)/MMSE at baseline × 100.

### 2.3 MR image processing

#### 2.3.1 MR image acquisition and structural preprocessing

T1‐weighted images for each participant were acquired using 3 Tesla scanners. Detailed information on the imaging protocols can be found in publicly accessible documents (https://adni.loni.usc.edu/methods/documents/mri‐protocols/). The T1‐weighted images were processed using FreeSurfer v7.4.1 (https://surfer.nmr.mgh.harvard.edu/), which involves multiple stages, including skull stripping, Talairach transformation, segmentation of subcortical structures, surface reconstruction, spherical registration, and cortical parcellation. We employed cross‐sectional and longitudinal processing streams to enhance the reliability of the measurement. In the cross‐sectional stream, each time point was processed independently, whereas the longitudinal stream generated an unbiased within‐subject template based on all the time points. This template, which enhances the reproducibility and lowers the variability in morphometric analysis, was used to initialize the additional longitudinal processing.^32^, ^33^, ^34^

#### 2.3.2 Hippocampal subfield segmentation and volume quantification

**FIGURE 2:**
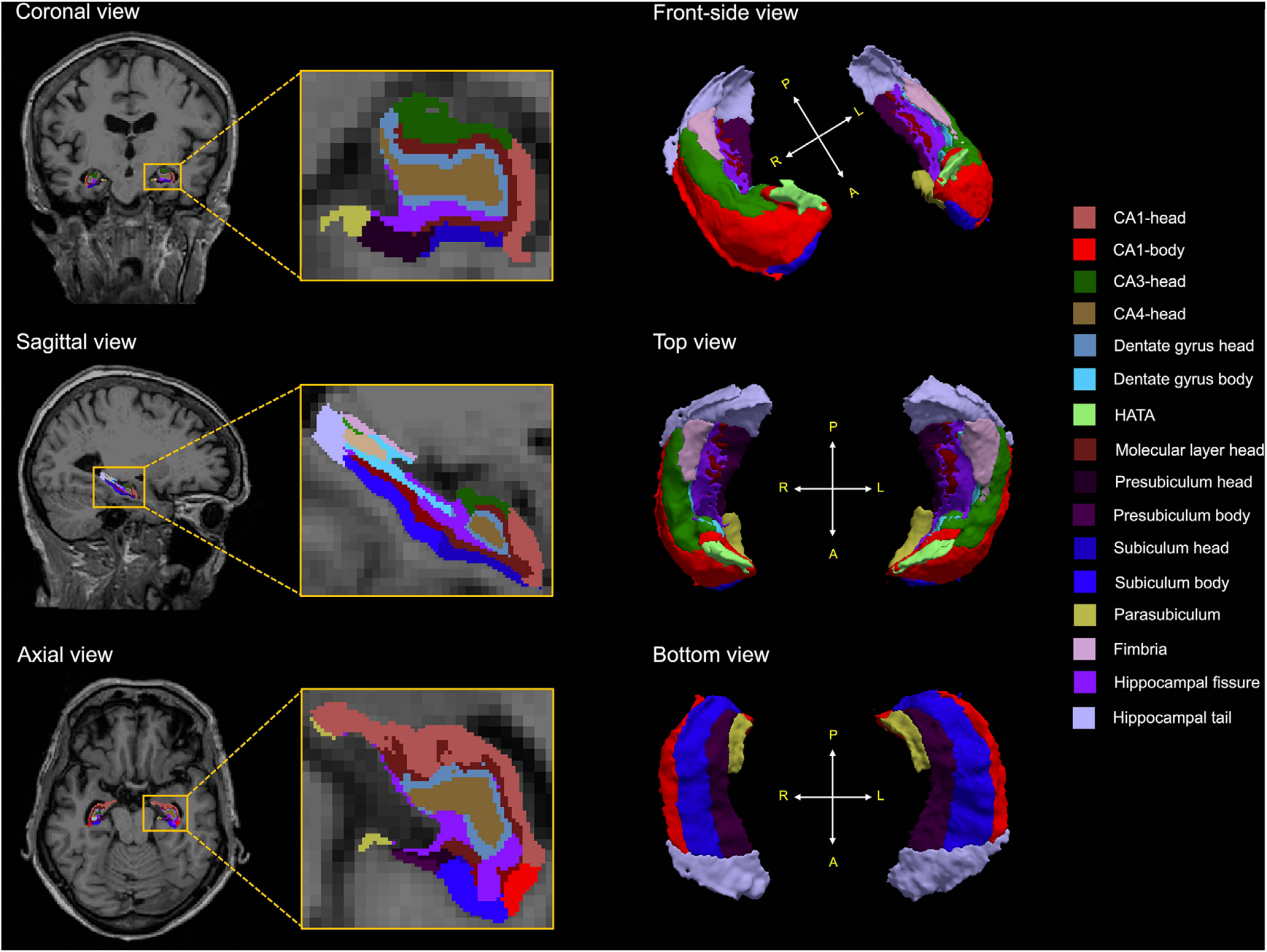
Hippocampal subfield segmentation in two‐dimensional and three‐dimensional views. The left panel shows representative coronal, sagittal, and axial slices with hippocampal subfields overlayed on T1‐weighted magnetic resonance images. The right panels present three‐dimensional surface renderings of the hippocampal subfields from the front, top, and bottom perspectives. The color coding corresponds to individual subfields, as shown in the legend. P, posterior; A, anterior; R, right; L, left; CA, cornu ammonis; HATA, hippocampal‐amygdala transition area.

Following the longitudinal preprocessing pipeline, hippocampal subfield segmentation was performed using an automated probabilistic atlas‐based approach integrated in FreeSurfer. This method defines the hippocampal substructures, including the CA1, CA3, CA4, subiculum, presubiculum, parasubiculum, dentate gyrus, molecular layer, hippocampal fissure, fimbria, and hippocampal‐amygdala transition area (HATA) using anatomical priors and Bayesian inference derived from ultra‐high‐resolution ex vivo MRI data.^35^ Figure 2 illustrates the results of hippocampal subfield segmentation.

To evaluate longitudinal structural changes, the volume of each segmented hippocampal subfield was extracted at baseline and during follow‐up using automated outputs generated by FreeSurfer following the segmentation process. Hippocampal subfield volume change was defined as the percentage difference between the baseline and follow‐up volumes over the 2‐year period: volume change (%) = (volume at follow‐up—volume at baseline)/volume at baseline × 100.

### 2.4 Construction and analysis of hippocampal subfield correlation networks

#### 2.4.1 Network modeling of inter‐subfield volumetric associations

To examine the structural relationships between the hippocampal subfields, we used MATLAB R2023a to calculate pairwise Pearson's partial correlation coefficients between the percentage volume changes of all subfield pairs across participants while controlling for baseline age, sex, education level, and APOE ε4 carrier status. A symmetric correlation matrix was generated to capture the inter‐subfield volumetric associations over the 2‐year period.

Significance‐based thresholding was used to ensure statistical rigor and reduce the possibility of spurious connections: only correlations with *p*‐values < 0.05 were retained. A sparse and comprehensible adjacency matrix was produced by setting all correlations that did not fit this condition to zero. Then, the matrix was used to construct an undirected, weighted network, where each node represented a hippocampal subfield, and the edge weights reflected the strength of statistically significant co‐atrophy patterns.

#### 2.4.2 Network‐based centrality analysis of hippocampal subfields

All centrality analyses were performed using MATLAB R2023a software. Four graph‐theoretical centrality metrics (degree centrality, betweenness centrality, closeness centrality, and eigenvector centrality) were calculated to evaluate the topological roles of the hippocampal subfields within the constructed network.

Degree centrality represents the number of direct connections a subfield has with other subfields based on significant pairwise correlations in volume change.^36^ The structural co‐variation of a subfield with high degree centrality with numerous other regions indicates its extensive involvement in the reconfiguration of the hippocampus. Equation (1) is the formula for degree centrality.^37^

(1) $$\;D_{i}=\sum_{j=1}^{N}a_{ij}$$

where $a_{ij}$ is an element of the adjacency matrix and $a_{ij}$ = 1 if a connection exists between nodes i and node j, and 0 otherwise.

Betweenness centrality measures the frequency with which a subfield resides on the shortest interaction paths between other subfields in the volume change network.^36^, ^38^ A high betweenness value indicates that the subfield might function as a structural link between multiple regions undergoing coordinated atrophy. Equation (2) is the formula for betweenness centrality.^36^

(2)

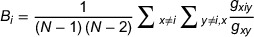

where $g_{xy}$ is the total number of the shortest geodesic paths between nodes x and y, and $g_{xiy}$ is the number of those paths that pass through node i.

Closeness centrality measures the overall accessibility of a subfield to all other subfields in a network. Structurally central, a subfield with high closeness centrality, enables efficient global atrophic signal transmission.^36^, ^39^ Equation (3) is the formula for closeness centrality.^37^

(3) $$\;C_{i}=\frac{N-1}{\sum_{j\neq i}l_{i,j}}$$

where $l_{i,j}$ is the shortest path distance between nodes i and j.

Eigenvector centrality considers connected subfields in addition to the network count. The high eigenvector centrality of a subfield related to other highly co‐varying subfields represents its integration within the core structural change network.^36^, ^40^ Equation (4) is a formula for eigenvector centrality.^36^

(4) $$\;E_{i}=\frac{1}{\lambda }\sum_{j=1}^{N}a_{ij}e_{j}$$

where λ is the largest eigenvalue of the adjacency matrix $A=[a_{ij}]$, and $e_{j}$ denotes the eigenvector component corresponding to node j.

### 2.5 Statistical analysis

All statistical analyses were performed using jamovi version 2.3.28.0 software and MATLAB R2023a. Longitudinal changes in general characteristics (such as cognitive scores) and hippocampal subfield volumes between the baseline and follow‐up were assessed using the paired *t*‐test.

The association between volume changes in individual hippocampal subfields and changes in cognitive score was examined using Pearson's partial correlation analysis, adjusting for baseline age, sex, education level, and APOE ε4 carrier status.

To examine interregional structural associations within the hippocampus, partial correlation coefficients were calculated between volume changes across all subfield pairs while adjusting for the same covariates. These pairwise correlations formed the basis for network construction.

For network modeling, statistically significant inter‐subfield correlations (*p* < 0.05) were retained to generate an adjacency matrix. An undirected, weighted graph was constructed in which nodes represent subfields and edges that reflect the strength and direction of co‐atrophy patterns.

For all analyses, statistical significance was set at *p* < 0.05.

### 2.6 Data availability

Data used for replication analyses were obtained from the ADNI database (adni.loni.usc.edu).^41^ The ADNI was launched in 2003 as a public–private partnership led by the principal investigator, Michael W. Weiner, MD. The primary goal of the ADNI is to test whether serial MRI, positron emission tomography, and other biological markers, as well as clinical and neuropsychological assessments, can be combined to measure the progression of MCI and early AD. For updated information, please refer www.adni‐info.org.

## 3 RESULTS

### 3.1 General demographics

Table 1 shows participants’ demographic information. A statistically significant decrease (mean change: –12.9%, *p* < 0.001) was observed in the MMSE score over the 2‐year period. The MMSE scores decreased from a mean score of 26.40 ± 1.93 at baseline to 22.90 ± 3.70 at the follow‐up, indicating substantial cognitive decline. The demographic characteristics of the excluded participants are provided in Table S1.

**TABLE 1:** Participants’ demographic characteristics and cognitive assessment scores.

Baseline age, mean (SD), y	75.1 (7.17)
Follow‐up interval, mean (SD), y	2.0 (0.15)
Sex	
Female, *n* (%)	102 (39.53)
Male, *n* (%)	156 (61.63)
Education (y)	
≤9, *n* (%)	4 (1.55)
10–12, *n* (%)	34 (13.18)
13–16, *n* (%)	120 (46.51)
>16, *n* (%)	100 (38.76)
APOE є4 carrier status	
(+/+), *n* (%)	41 (15.89)
(±), *n* (%)	123 (47.68)
(–/–), *n* (%)	94 (36.43)
ΔMMSE, mean (SD), %	−12.9 (13.8)

Abbreviations: APOE, apolipoprotein; MMSE, Mini‐Mental State Examination; N.A., not applicable; SD, standard deviation.

### 3.2 Longitudinal differences in hippocampal subfield volume

**FIGURE 3:**
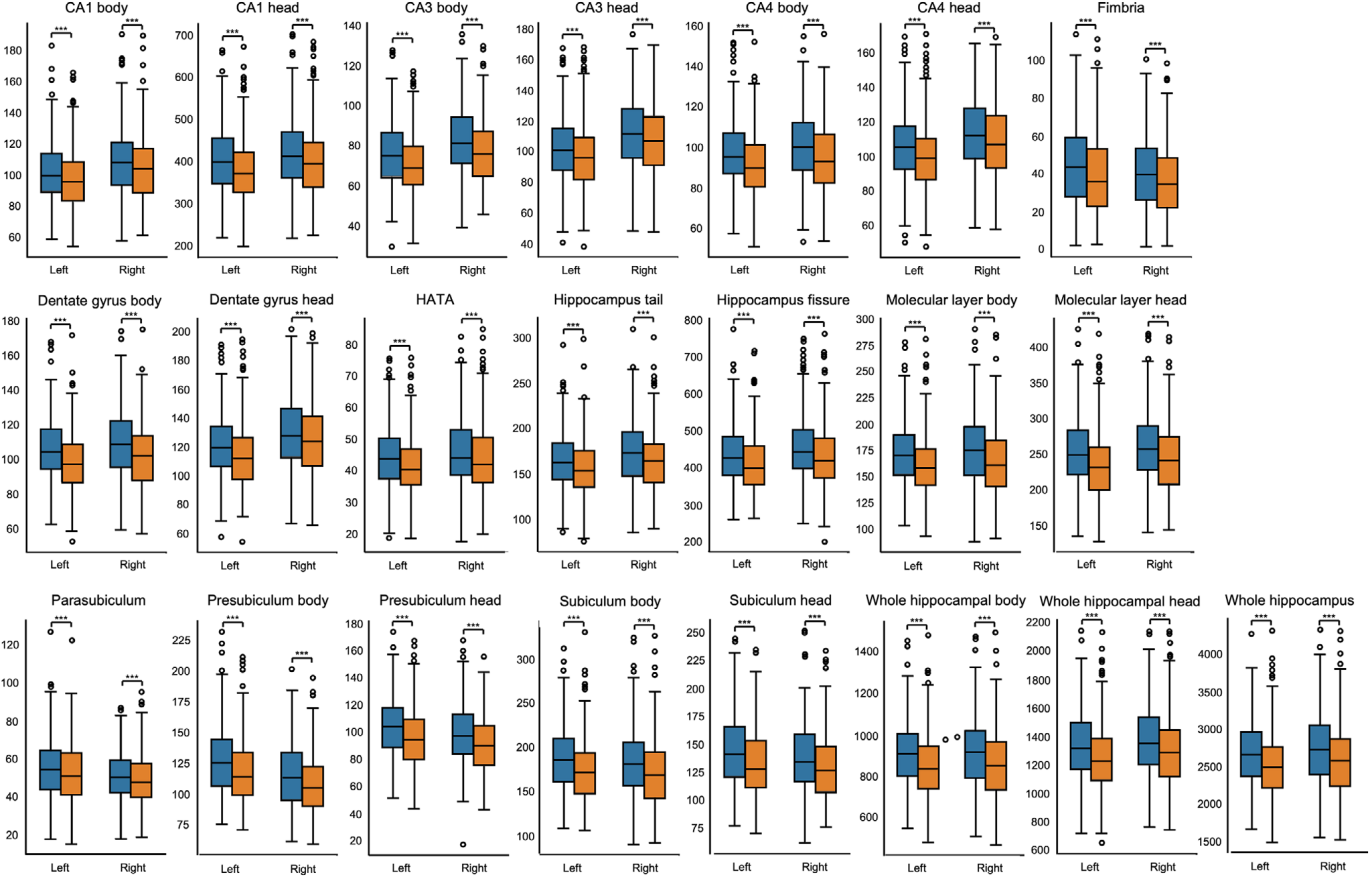
Longitudinal changes in hippocampal subfield volumes between baseline and follow‐up. The boxplots represent hippocampal subfield volumes at baseline (blue) and 2‐year follow‐up (orange) for the left and right hemispheres. Significant *p*‐values are marked with ^*^ *p* < 0.05, ^**^ *p* < 0.01, ^***^ *p* < 0.001. CA, cornu ammonis; HATA, hippocampal‐amygdala transition area.

**FIGURE 4:**
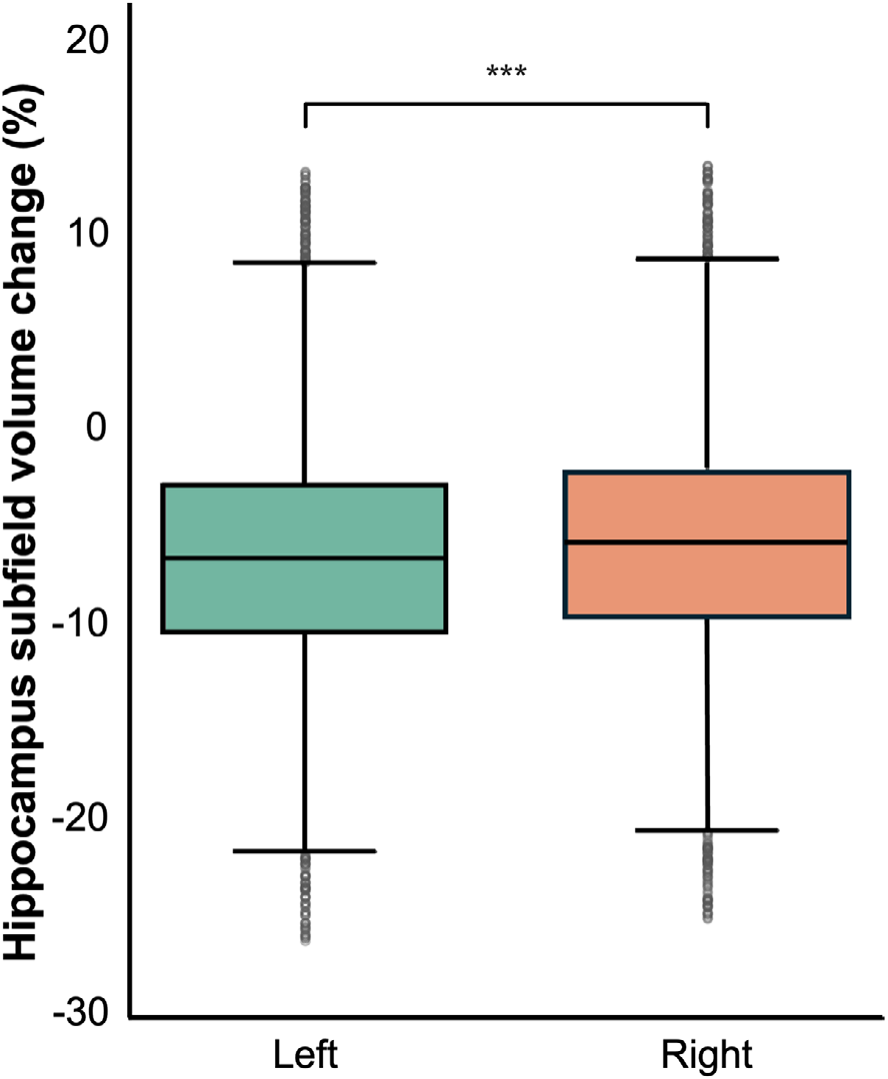
Comparison of average left and right hippocampal subfield volume change over the 2‐year period. Significant *p*‐values are marked with ^*^ *p* < 0.05, ^**^ *p* < 0.01, ^***^ *p* < 0.001.

Paired comparisons between the baseline and 2‐year follow‐up revealed a significant volume decrease in multiple hippocampal subfields (Table S2). A significant longitudinal decrease in volume was observed in all hippocampal subfields (*p* < 0.001). The largest percent volume decrease was observed in the left fimbria (mean change: –9.11%, *p* < 0.001), left presubiculum head (mean change: –7.99%, *p* < 0.001), left subiculum head (mean change: –7.93%, *p* < 0.001), right CA3 body (mean change: –7.07%, *p* < 0.001), and left CA3 body (mean change: –7.03%, *p* < 0.001). Figure 3 presents boxplots of the hippocampal subfield volumes at baseline and 2‐year follow‐up, separately for the left and right hemispheres. A significant decrease in the median volume was observed across most subfields at follow‐up, indicating longitudinal atrophy. Figure 4 compares the average percent volume change between the left and right subfields to further examine hemispheric asymmetry. The left and right hippocampus decreased by an average of 6.35% and 5.47%, respectively. Both hemispheres exhibited consistent atrophy patterns; however, the left hippocampus demonstrated a significantly greater volume decrease overall (*p* < 0.001).

### 3.3 Longitudinal association between hippocampal subfield volume change and cognitive decline

**FIGURE 5:**
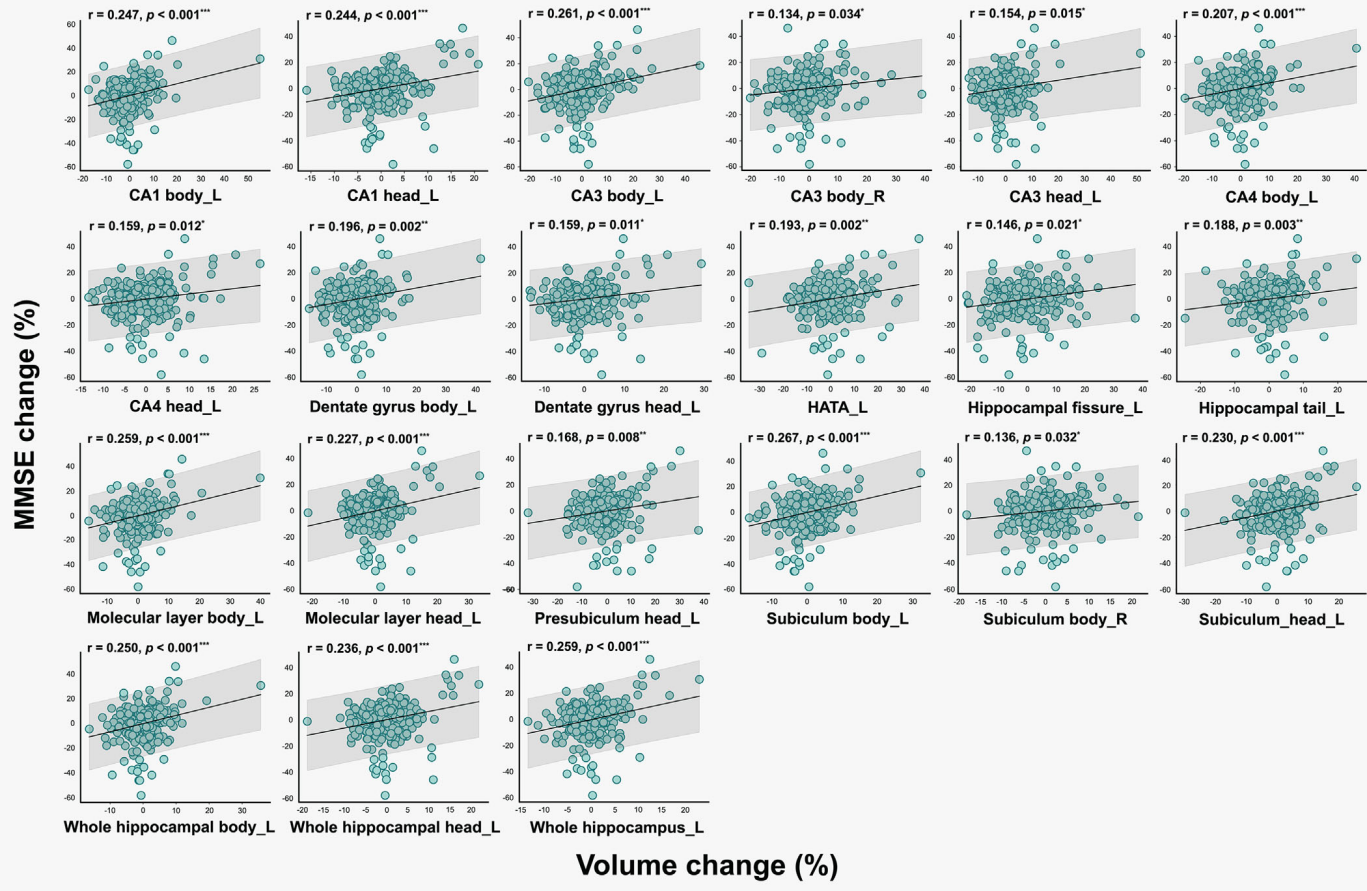
Significant association of hippocampal subfield volume changes with change in MMSE scores. The Pearson's *r* (*r*) and *p*‐values were calculated using a partial correlation, considering all participants and controlling for factors such as baseline age, sex, education, and apolipoprotein e4 carrier status. Significant *p*‐values are marked with ^*^ *p* < 0.05, ^**^ *p* < 0.01, ^***^ *p* < 0.001. R, right; L, left; CA, cornu ammonis; HATA, hippocampal‐amygdala transition area.

Partial correlation analysis revealed statistically significant associations between volume changes in specific hippocampal subfields and changes in MMSE scores over the 2‐year period (Table 2). Of the left hippocampal subfields, the subiculum body (*r* = 0.267, *p* < 0.001), CA3 body (*r* = 0.261, *p* < 0.001), molecular layer body (*r *= 0.259, *p* < 0.001), and whole hippocampus (*r* = 0.259, *p *< 0.001) showed strong positive correlation with changes in MMSE scores. In the right hippocampus, the CA3 body (*r* = 0.134, *p* = 0.034) and subiculum body (*r* = 0.136, *p* = 0.032) showed a significant positive correlation. Figure 5 presents the hippocampal subfields that showed statistically significant partial correlations between volume changes and changes in MMSE scores over the 2‐year period. The correlation results between changes in MMSE scores and volume changes for all hippocampal subfields are provided in Table S3.

**TABLE 2:** Significant associations between hippocampal subfield volume changes and changes in MMSE scores.

Hippocampal subfield	Statistical results	
	Pearson's *r*	*p*‐value
CA1 body L	0.247	<0.001^***^
CA1 head L	0.244	<0.001^***^
CA3 body L	0.261	<0.001^***^
CA3 body R	0.134	0.034^*^
CA3 head L	0.154	0.015^*^
CA4 body L	0.207	<0.001^***^
CA4 head L	0.159	0.012^*^
Dentate gyrus body L	0.196	0.002^**^
Dentate gyrus head L	0.159	0.011^*^
HATA L	0.193	0.002^**^
Hippocampal tail L	0.146	0.021^*^
Hippocampal fissure L	0.188	0.003^**^
Molecular layer body L	0.259	<0.001^***^
Molecular layer head L	0.227	<0.001^***^
Presubiculum head L	0.168	0.008^**^
Subiculum body L	0.267	<0.001^***^
Subiculum body R	0.136	0.032^*^
Subiculum head L	0.230	<0.001^***^
Whole hippocampal body L	0.250	<0.001^***^
Whole hippocampal head L	0.236	<0.001^***^
Whole hippocampus L	0.259	<0.001^***^

*Note*: The Pearson's *r* and *p*‐values were calculated using a partial correlation, considering all participants and controlling for factors such as baseline age, sex, education, and apolipoprotein e4 carrier status. Significant *p*‐values are marked with ^*^ *p* < 0.05, ^**^ *p* < 0.01, ^***^ *p* < 0.001.

Abbreviations: CA, cornu ammonis; HATA, hippocampal‐amygdala transition area; L, left; R, right.

### 3.4 Inter‐regional associations of hippocampal subfield volume changes

**FIGURE 6:**
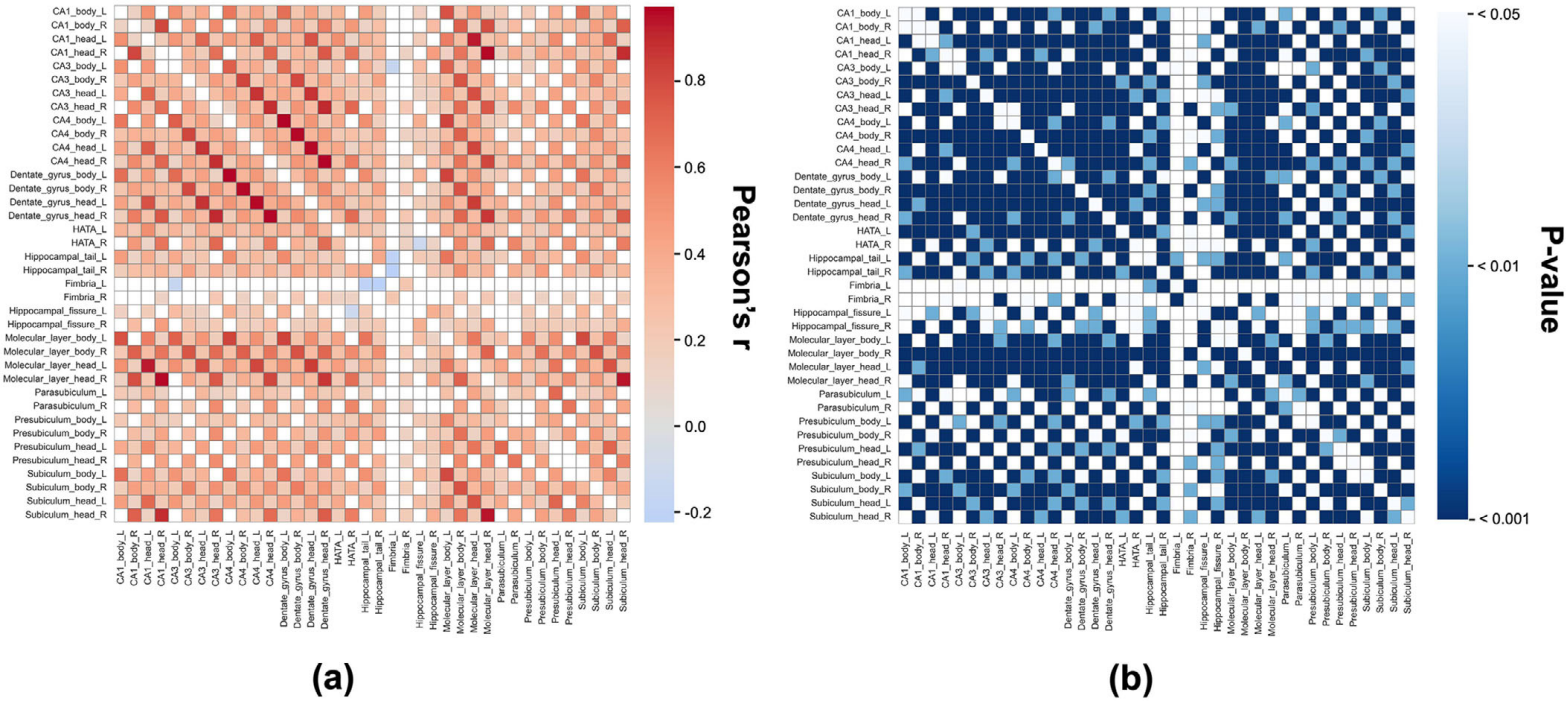
Heatmaps of inter‐subfield correlation in hippocampal volume change: (a) Pearson's *r* and (b) *p*‐values. R, right; L, left; CA, cornu ammonis; HATA, hippocampal‐amygdala transition area.

Interregional associations between volume changes in hippocampal subfield pairs were calculated using Pearson's partial correlation. Figure 6a presents a heatmap of the resulting correlation matrix (*r*), while Figure 6b shows the corresponding *p*‐values. The strongest positive inter‐subfield correlations were observed between the left CA4 body and left dentate gyrus body (*r* = 0.972, *p* < 0.001), right CA4 head and right dentate gyrus body (*r* = 0.963, *p* < 0.001), right CA4 body and right dentate gyrus body (*r* = 0.959, *p* < 0.001), right CA1 head and right molecular layer head (*r* = 0.959, *p* < 0.001), and left CA4 head and left dentate gyrus head (ρ = 0.957, *p* < 0.001). The complete pairwise correlation values and corresponding *p*‐values for all subfield pairs are provided in Table S4.

### 3.5 Network‐based centrality analysis of hippocampal subfield volume changes

**FIGURE 7:**
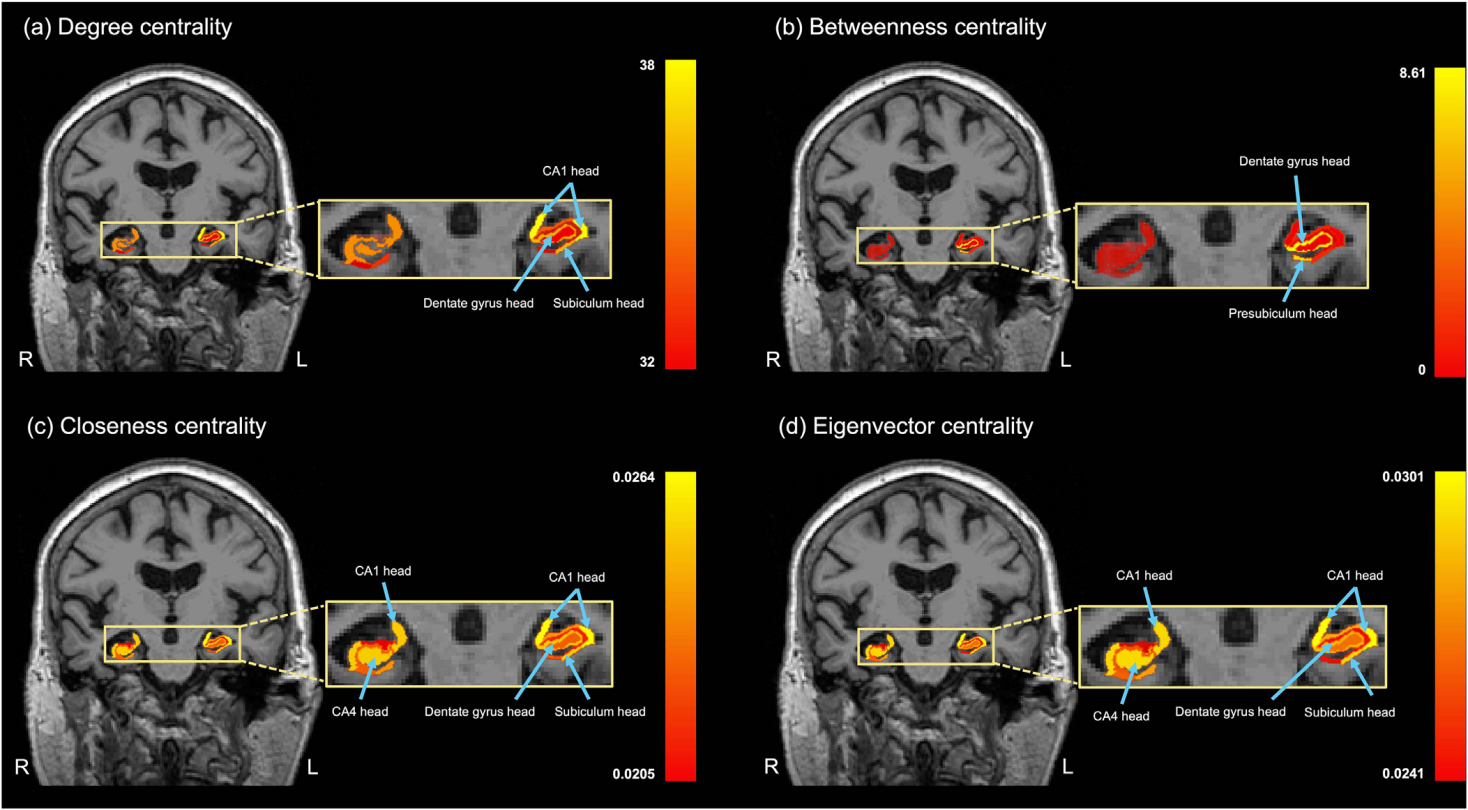
Visualization of hippocampal subfield centrality measures in the structural correlation network. Representative coronal images are shown for each centrality metric: (a) degree centrality, (b) betweenness centrality, (c) closeness centrality, and (d) eigenvector centrality. Yellow indicates higher centrality values, and red indicates lower values. R, right; L, left; CA, cornu ammonis.

Network centrality measures were calculated to evaluate the relative significance of volume changes in the hippocampal subfields within the structural correlation network of volumetric alterations. Table 3 presents the ranks of degree, betweenness, closeness, and eigenvector centrality per subfield. Figure 7 displays each centrality metric mapped onto the hippocampal anatomical views, with yellow indicating higher centrality values.

**TABLE 3:** Network centrality measures of hippocampal subfields in the structural correlation network.

Subfield	Degree	Betweenness	Closeness	Eigenvector
CA1 body L	32	1.29556	0.02273	0.02588
CA1 body R	33	1.30625	0.02326	0.02687
CA1 head L	38	4.03125	0.02632	0.03008
CA1 head R	37	3.00362	0.02564	0.02956
CA3 body L	31	0.93946	0.02222	0.02522
CA3 body R	34	2.25991	0.02381	0.02727
CA3 head L	36	2.35723	0.02500	0.02896
CA3 head R	33	1.78503	0.02326	0.02649
CA4 body L	35	2.62817	0.02439	0.02806
CA4 body R	35	2.42821	0.02439	0.02810
CA4 head L	35	1.92806	0.02439	0.02827
CA4 head R	37	3.00362	0.02564	0.02956
Dentate gyrus body L	36	0.16146	0.01471	0.00476
Dentate gyrus body R	36	3.93172	0.01887	0.01750
Dentate gyrus head L	37	8.42817	0.02500	0.02820
Dentate gyrus head R	37	3.29920	0.02500	0.02863
HATA_L	35	8.60723	0.02564	0.02911
HATA_R	31	3.00362	0.02564	0.02956
Hippocampal tail L	34	1.98413	0.02439	0.02827
Hippocampal tail R	35	2.42606	0.02222	0.02445
Fimbria L	8	2.11715	0.02381	0.02732
Fimbria R	23	2.61051	0.02439	0.02797
Hippocampal fissure L	22	0.41920	0.01852	0.01724
Hippocampal fissure R	29	1.10466	0.02128	0.02329
Molecular layer body L	36	2.61964	0.02500	0.02886
Molecular layer body R	36	3.33967	0.02564	0.02949
Molecular layer head L	37	3.00362	0.02564	0.02956
Molecular layer head R	37	2.52773	0.02500	0.02890
Parasubiculum L	28	4.44433	0.02083	0.02205
Parasubiculum R	29	1.30145	0.02128	0.02306
Presubiculum body L	33	2.61342	0.02326	0.02620
Presubiculum body R	25	1.06362	0.01961	0.01955
Presubiculum head L	35	7.78895	0.02439	0.02733
Presubiculum head R	36	2.57570	0.02500	0.02886
Subiculum body L	36	8.07489	0.02500	0.02837
Subiculum body R	37	3.00362	0.02564	0.02956
Subiculum head L	37	3.33967	0.02564	0.02949
Subiculum head R	35	2.24421	0.02439	0.02811

Abbreviations: CA, cornu ammonis; HATA, hippocampal‐amygdala transition area; L, left; R, right.

The five leading subfields for degree centrality were the left CA1 head, left dentate gyrus head, left molecular layer head, left subiculum head, and right subiculum body. The left CA1 head, left dentate gyrus head, and left subiculum head showed an increased degree of centrality in the anterior subfields of the left hippocampus (Figure 7a).

The highest betweenness centrality values were observed in the left dentate gyrus head and body, left subiculum body, left presubiculum head, and left parasubiculum, predominantly involving the anterior and intermediate subfields of the left hippocampus (Figure 7b).

Regarding the closeness centrality, the most central subfields were the left CA1 head, left dentate gyrus head, left molecular layer head, left subiculum head, and right subiculum body. The left and right hippocampus consistently showed a high closeness centrality in the CA1 head (Figure 7c). Additional subfields with elevated values included the right subiculum body, right CA4 head, right dentate gyrus head, and right molecular layer head.

The five subfields with the highest eigenvector centralities were the left CA1 head, right subiculum body, right CA1 head, right CA4 head, and right dentate gyrus head. Notably, the left CA1 head exhibited the most prominent eigenvector centrality, indicating its role as a key hub within the network. Elevated eigenvector centrality was also observed across several regions of the right hippocampus, particularly the CA1 head, CA4 head, dentate gyrus head, and subiculum body (Figure 7d).

Notably, the left CA1 head, left dentate gyrus head, left molecular layer head, and left subiculum head consistently ranked among the top five subfields across all four‐centrality metrics. This convergence highlights these regions as core hubs within the hippocampal subfield network.

## 4 DISCUSSION

In this study, we investigated the longitudinal hippocampal subfield volume changes in individuals who progressed from MCI to AD over the 2‐year period and examined their associations with cognition and structural network properties.

Longitudinal analysis revealed extensive and significant volume loss in almost all hippocampal subfield regions over the 2‐year follow‐up period. This pattern of diffuse atrophy is consistent with previous studies indicating that AD begins in multiple hippocampal subfields, particularly the CA1, subiculum, fimbria, molecular layer, and dentate gyrus, and progresses to other subfields in a gradual degenerative process.^18^, ^42^ In this study, significant volume decreases were observed in the fimbria, presubiculum, and subiculum. Reportedly, the fimbria, a white matter bundle in the hippocampus, has been suggested to exhibit microstructural alterations in AD, including changes potentially related to demyelination and iron accumulation, which may contribute to its vulnerability to atrophy.^43^, ^44^ The significant atrophy of the presubiculum and subiculum observed in this study is consistent with numerous studies reporting that atrophy in this region is associated with NFTs and synaptic destruction in AD pathology.^45^, ^46^, ^47^ Atrophy in these regions leads to memory impairment and overall cognitive decline, with subiculum atrophy specifically causing impairments in episodic memory and spatial memory.^48^ In addition, volume decreases were significantly greater in the left hippocampus than in the right hippocampus. This lateralization is consistent with prior findings and may reflect language memory dysfunction in early AD.^49^ This asymmetric vulnerability suggests that the left hippocampus plays a more prominent role in the initial structural breakdown associated with the progression of AD.

A significant longitudinal association was observed between cognitive decline, as measured by hippocampal subfield volume changes and changes in MMSE scores. Strong correlations were observed in the left subiculum body, left CA3 body, and left molecular layer body. The predominance of significant associations in the left hippocampus highlights the lateralized vulnerability of the hippocampus to cognitive decline, as repeatedly reported in previous studies.^50^, ^51^ The left CA3 is involved in the encoding and discrimination of new information, and its atrophy may impair neuroplasticity maintenance, accelerate disease progression, and thereby induce cognitive decline.^52^, ^53^, ^54^ Therefore, these results suggest that atrophy in specific subfields may be a primary indicator of cognitive decline.

The inter‐regional association of hippocampal subfield volume changes demonstrated a highly structured and anatomically consistent pattern of covariance, emphasizing that the hippocampus is not a collection of isolated subunits but rather a set of subfields showing coordinated structural covariance patterns. This result is consistent with those of previous studies, suggesting that hippocampal subfields interact through specialized circuits supporting various memory and learning processes.^29^, ^55^, ^56^ Strong positive correlations were observed between anatomically and functionally connected regions, such as the CA1, CA3, and CA4. These relationships are anatomically consistent with the canonical trisynaptic pathway, although the present analysis reflects coordinated volumetric change rather than direct circuit‐level connectivity.^57^, ^58^ These volume change relationships between regions likely reflect common vulnerabilities to AD‐related neurodegeneration and atrophy. The most prominently interconnected subfields were centered on CA‐dentate gyrus system, highlighting its role as a core structural unit in hippocampal degeneration. In particular, strong and widespread associations involving anterior hippocampal subfields, including the CA1 and dentate gyrus, suggest tightly coordinated atrophy during disease progression. Given their established roles in information encoding, pattern separation, and early vulnerability to AD pathology, the prominence of these regions supports the notion that degeneration within the hippocampus is organized around functionally critical subfield systems rather than isolated regions. In addition, several output‐related subfields, including subiculum, presubiculum, and molecular layer, exhibited extensive inter‐subfield associations, indicating their integrative role in hippocampal network degeneration.^54^, ^59^ These regions constitute major relay pathways between the hippocampus and cortical structures such as the entorhinal cortex, and their coordinated structural changes may reflect a system‐level reorganization of hippocampal output circuitry in AD.^60^ The involvement of these subfields in extensive associations supports the concept that hippocampal atrophy in AD is not spatially random but follows organized anatomical and functional gradients.^61^ In addition, most of the strong correlations occurred within the same hemisphere, particularly within the left hippocampus. The left dentate gyrus head and left CA4 head showed the strongest and most consistent associations with other left hippocampal subfields, indicating tightly coupled anterior hippocampal degeneration. Similar patterns were observed in the right hippocampus, albeit to a weaker extent. This hemispheric modularity may reflect localized vulnerability and structural plasticity mechanisms. Thus, mapping inter‐subfield dependencies may help characterize coordinated patterns of atrophic change in AD, although such patterns should not be interpreted as direct evidence of functional or anatomical connectivity. It is important to note that the structural covariance networks used in this study are derived from volumetric changes observed in T1‐weighted MRI, and therefore do not reflect microstructural degeneration or functional connectivity directly. As such, interpretations of centrality and inter‐regional associations must be limited to morphometric covariance.

Based on a network‐based centrality analysis of volume change patterns over the 2‐year longitudinal period, a set of hippocampal subfields rather than a single region consistently occupied central positions within the structural covariance network. This finding suggests that they function as organizational hubs in hippocampal degeneration as a result of prominent and coordinated atrophy. Subfields such as the left CA1 and dentate gyrus consistently ranked high across centrality measures. Their centrality reflects both high structural importance and coordination with widespread atrophy. In particular, the high degree of centrality of the left CA1 head and left dentate gyrus head indicated that their volume reduction patterns over time were closely aligned with those of many other subfields. These regions are also known for early pathological changes, such as synaptic loss and tau accumulation.^62^, ^63^ In the change‐based network, their centrality highlights their vulnerability and influence on the formation of downstream degeneration patterns. High betweenness centrality in subfields, such as the left subiculum body and left presubiculum head, suggests that these subfields may act as transitional intermediaries within the structural covariance network, mediating atrophic co‐variation between subfields that otherwise show weaker covariance. This may reflect their anatomical role in connecting hippocampal circuits with cortical outputs, particularly the entorhinal cortex.^64^ Subfields with high closeness and eigenvector centrality, such as the molecular layer head and subiculum head, may play a more integrated role by being involved in widespread co‐atrophy patterns. This suggests that changes in these regions may indicate overall hippocampal degeneration and potentially serve as a summary indicator of network‐wide degeneration.^65^ By capturing simultaneous atrophy, this analysis identifies how subfields influence or reflect degeneration in connected regions.^66^ From the perspective of structural volume covariance networks, regions with high centrality are thought to represent hubs that are particularly vulnerable to disease‐related degeneration due to their extensive inter‐regional coupling. Previous studies have shown that highly central nodes within covariance networks are more susceptible to pathological burden and may reflect regions that are particularly susceptible to coordinated neurodegeneration.^67^, ^68^, ^69^ In particular, Chu et al. demonstrated altered hippocampal subfield volume covariance patterns and network properties in AD using a cross‐sectional design.^70^ However, cross‐sectional covariance analyses are inherently limited in their ability to capture the temporal dynamics of coordinated neurodegeneration, as they reflect inter‐individual variability at a single time point rather than within‐subject change. In this context, the present longitudinal analysis extends prior work by constructing structural covariance networks based on within‐subject volume changes over time, allowing direct assessment of coordinated atrophic progression. The identification of the left CA1 head and dentate gyrus head as central hubs in this change‐based network suggests that coordinated atrophy in these subfields reflects a network‐driven degeneration process unfolding over time, rather than isolated regional vulnerability.

This study provides new insights into the structural degeneration patterns of the hippocampus during the progression of AD. The primary strength of this approach is the detailed segmentation of the hippocampus into subfields such as “CA1 head,” “CA1 body,” “subiculum head,” and “subiculum body,” rather than using whole‐region labels like “CA1” or “subiculum.” This allowed us to detect subtle yet meaningful regional changes in the structural interactions. A notable finding was the severe atrophy in the left fimbria, showing the greatest volume reduction over 2 years. Interestingly, the left fimbria exhibited significant atrophy but weak network integration, suggesting a distinct degeneration trajectory or early disconnection from hippocampal circuitry, indicating a need for further study.^71^ Unlike prior studies using functional MRI (fMRI)‐based connectivity, we constructed a network based on the correlation between changes in hippocampal subfield volume over 2 years and conducted analyses grounded in longitudinal structural covariance. This approach provides a time‐based map of the co‐degeneration of hippocampal subfields. This study highlights the potential of network information‐based morphological analysis for understanding AD progression.

However, this study had some limitations. First, although the sample size was relatively large, all participants were drawn from a single well‐characterized cohort, which may limit the generalizability of the findings to broader or more heterogeneous populations. Future studies, including independent cohorts or population‐based samples, would be valuable to validate the reproducibility of the observed hippocampal subfield network patterns. Second, a difference in sex distribution was observed between included and excluded participants, with a higher proportion of males in the included cohort. Although sex was accounted for in the statistical analyses, this imbalance may introduce residual selection bias and should be considered when interpreting the results. Third, hippocampal subfield volumes were obtained using FreeSurfer's automated segmentation, which may result in misclassification or inconsistencies in boundaries. Therefore, further studies should consider cross‐validation with alternative segmentation tools to improve anatomical accuracy. Finally, future studies incorporating multimodal imaging such as diffusion tensor imaging or fMRI may further elucidate the microstructural and functional aspects of hippocampal degeneration that cannot be captured using structural MRI alone.

## 5 CONCLUSION

In this study, we quantitatively examined longitudinal changes in hippocampal subfield volume in individuals who progressed from MCI to AD over the 2‐year period. This study demonstrated that hippocampal subfield atrophy during AD progression follows a spatially structured and interconnected pattern. Using high‐resolution subfield segmentation and network‐based analysis of longitudinal volume changes, we identified anatomically and functionally central subfields that could serve as potential imaging biomarkers of disease progression. These findings emphasize the importance of considering individual regions and their interdependencies and offer new insights into the network‐level organization of hippocampal degeneration in AD.

## CONFLICT OF INTEREST STATEMENT

The authors declare no conflicts of interest.

## Supporting information



Supporting Information

Supporting Information

Supporting Information

Supporting Information

## Data Availability

The data that support the findings of this study were obtained from the Alzheimer's Disease Neuroimaging Initiative (ADNI) database (adni.loni.usc.edu). Access to ADNI data requires registration and approval by the ADNI Data Sharing and Publications Committee.
